# Identify. Quantify. Predict. Why Immunologists Should Widely Use Molecular Imaging for Coronavirus Disease 2019

**DOI:** 10.3389/fimmu.2021.568959

**Published:** 2021-05-13

**Authors:** Freimut D. Juengling, Antonio Maldonado, Frank Wuest, Thomas H. Schindler

**Affiliations:** ^1^ Medical Faculty, University Bern, Bern, Switzerland; ^2^ Department of Oncology, Faculty of Medicine and Dentistry, University of Alberta, Edmonton, AB, Canada; ^3^ Department of Nuclear Medicine and Molecular Imaging, Quironsalud Madrid University Hospital, Madrid, Spain; ^4^ Mallinckrodt Institute of Radiology, Division of Nuclear Medicine, Washington University School of Medicine, Saint Louis, MO, United States

**Keywords:** SARS-CoV-2, CXCR4, P2X7 (purino) receptor, ACE-2-receptor, COX-2, cytokine storm, molecular imaging, COVID-19

## Abstract

Molecular imaging using PET/CT or PET/MRI has evolved from an experimental imaging modality at its inception in 1972 to an integral component of diagnostic procedures in oncology, and, to lesser extent, in cardiology and neurology, by successfully offering *in-vivo* imaging and quantitation of key pathophysiological targets or molecular signatures, such as glucose metabolism in cancerous disease. Apart from metabolism probes, novel radiolabeled peptide and antibody PET tracers, including radiolabeled monoclonal antibodies (mAbs) have entered the clinical arena, providing the *in-vivo* capability to collect target-specific quantitative *in-vivo* data on cellular and molecular pathomechanisms on a whole-body scale, and eventually, extract imaging biomarkers possibly serving as prognostic indicators. The success of molecular imaging in mapping disease severity on a whole-body scale, and directing targeted therapies in oncology possibly could translate to the management of Coronavirus Disease 2019 (COVID-19), by identifying, localizing, and quantifying involvement of different immune mediated responses to the infection with SARS-COV2 during the course of acute infection and possible, chronic courses with long-term effects on specific organs. The authors summarize current knowledge for medical imaging in COVID-19 in general with a focus on molecular imaging technology and provide a perspective for immunologists interested in molecular imaging research using validated and immediately available molecular probes, as well as possible future targets, highlighting key targets for tailored treatment approaches as brought up by key opinion leaders.

## Introduction

In response to the current pandemic of Coronavirus Disease 2019, medical imaging research first focused on procedure and safety guidelines and the integration of medical imaging into the diagnostic workflow for COVID-19 (1–4). In the meantime, the spectrum of associated symptoms attributed to the disease has become larger, clearly exceeding the notion of it being primarily a severe pneumonia with fever, cough and dyspnea, thus warranting to extend medical imaging to provide information not only on pulmonary condition. Early observational studies reported older age and the presence of comorbidities as risk factors for increased disease severity. With increasing incidence of COVID-19, an increasing number of severe courses of disease have been reported in younger patients with no pre-existing, medical conditions (5). Acute respiratory distress syndrome (ARDS) is a common complication of severe viral pneumonia, and is reported to occur in 15.6% of patients with severe COVID-19 pneumonia (6).

Accumulating evidence suggests that some of the detrimental effects seen in patients with COVID-19 is attributed to an overly host antiviral defense as described earlier in severe acute respiratory syndrome (SARS), leading to hyperinflammatory reactions (7) or cytokine storm syndrome, sometimes culminating to affect the central nervous system (CNS) (8), and often associated with increased serum levels of several inflammatory cytokines and chemokines (9).

There is also an increasing number of COVID-19 associated cases with systemic or localized inflammatory disease, such as vasculitis (10), endothelial cell dysfunction (11), Kawasaki-like disease in the young (12), nephritis (13), Guillain–Barré-syndrome (14) or inflammatory bowel disease (15), with no indication of lab findings, clinical signs, or symptoms predictive of their appearance. The factors that ultimately trigger severe illness in individuals infected with SARS-CoV-2 are not completely understood and as in other systemic inflammatory conditions, the development of severe disease may be preceded by signs of locally contained inflammatory reactions, occult to clinical observation, but possibly visible by molecular imaging.

In a recent comment, Merad et al. appealed “to start measuring longitudinally and with as much granularity as possible the systemic and potentially local inflammatory responses induced by SARS- CoV-2 and how these pathways are modulated by the different therapies currently used as standard of care or in clinical trials, [ … ] clinical centers that have the means to perform deep-monitoring studies now have the responsibility to conduct such studies and to share their strategies and results with the scientific community”. In this article, the authors present their position on how molecular imaging for COVID-19 fits to this appeal and why it should widely be used by immunologists.

## General Clinical Contribution of Medical Imaging to Longitudinally Investigate COVID-19

Medical imaging, in certain, clinical settings being on the frontline of diagnostics in COVID-19 (4, 16, 17), is able to provide objective, longitudinal measures of both systemic and local disease involvement. During the unfolding of the pandemic, chest-CT scanning has grown into a clinically well-established procedure in the early diagnosis of COVID-19 in patients with symptoms ranging from none to very severe (3). Depending on the diagnostic workflow of an individual institution, CT may well be a very initial diagnostic procedure, in some situations even preceding PCR diagnostics (18, 19), and accordingly, CT has been integrated into various diagnostic algorithms for COVID-19 (4, 20). Recent progress in clinical applications of artificial intelligence (AI) in COVID-19 (21–23) has led to approval of first clinical products for AI-based, automated detection of COVID-19 related changes in chest-CT, integrating differentiation criteria not always obvious to the eye of less experienced observers.

By current recommendations of the American and European Radiological Societies, computed tomography (CT) of the chest is indicated in COVID-19 patients with respiratory symptoms and may be helpful in patients with milder symptoms but co-morbidities, with repeat CTs indicated in cases with suspected complications (*e.g.* superinfection, pulmonary embolism) (24).

With systematic data just being built up, however, it still remains fluent, to which extent imaging studies add to the clinical management of COVID-19 and its complications. Current notion includes that specific exams might add to clinical understanding in selected cases as in suspected myositis, perimyocarditis or possible CNS involvement (25, 26).

As a subgroup of COVID-19 patients that seems to do well after getting out of the intensive care unit, dies of acute respiratory syndrome just several days later, without clinical signs indicating their imminent deterioration, systematic whole-body imaging data on the inflammatory burden during the course of disease, including a refractory phase after intensive care, could be a potential life-saver for individual patients.

## Rationale for and Potential of Systematic Clinical Studies on Targeted Molecular Imaging Using Positron Emission Tomography in COVID-19

The scientific response to COVID-19 has been extraordinary, with a plethora of studies designed to rapidly unravel the pathogenesis of COVID-19 and potential therapeutic strategies, often posted in preprint servers to speed up access to the scientific community. While preclinical in-vitro and in-vivo data has rapidly accumulated and undoubtedly plays an important role in anticipation and design of clinical studies and future, prospective clinical trials, the need for human *in-vivo*-evidence for the applicability of proposed therapeutic strategies is still unmet and evolving.

MRI and PET/CT are the two key imaging modalities that have been investigated extensively for providing non-invasive information on localization and extent of inflammatory disease and disease of autoimmune origin (27, 28), with MRI being the most frequently used modality and whole-body PET/CT yielding the highest sensitivity and specificity even for clinically occult disease [for review (29)].

The exquisite sensitivity of PET imaging is based on the tracer principle, where radiopharmaceuticals designed to specifically aim a physiological target are introduced into the patient and are subsequently detected by quantitative counting of positron emissions originated by their radioligand, allowing for localization and visualization by computed reconstruction of their spatial distribution. PET thus allows for observing and quantifying molecular signatures as targeted by the radiopharmaceutical, non-invasively, *in vivo*, with a resolution in the space domain in the range of millimeters and in the time domain in the range of seconds to a couple of days or even weeks, depending on the specific half-life of the radionuclide coupled to the carrier molecule.

As defined by the tracer principle, the amount of carrier-target-complex injected is always below the threshold of a pharmacological effect, with a sensitivity of the method allowing for quantitation of picomolar to nanomolar concentrations (30). The technological evolution of combining advanced PET detectors and CT into one single hybrid scanner has led to scanning times down from an initial hour-long procedure to whole-body scans feasible in a couple of seconds, having allowed a widespread clinical adoption of PET for oncologic indications and facilitating imaging procedures even in the severely ill. The most frequently used PET radiopharmaceutical to date is 18-F-Fluorodeoxyglucose (FDG), targeting the glucose transporter 1 (GLUT-1), which is overexpressed in many malignant cells, but also in cells responsible for inflammatory response.

The increased availability of the technology over the past two and a half decades of its clinical implementation has also broadened its clinical applications, and imaging of inflammatory disease, *e.g*. in large vessel vasculitis, autoimmune encephalitis or fever without origin, are now established indications for FDG-PET/CT (29, 31–33). More recently, hybrid molecular imaging using FDG-PET/MRI has attracted attention for combining the superb imaging characteristics of both, MRI and PET in a simultaneous exam, defining a new imaging gold standard (34) with increasing sensitivity and specificity in soft tissue pathologies.

Early case series in COVID-19 patients indicate that whole-body FDG-PET/CT is highly sensitive for detection and quantification of inflammatory pulmonary involvement (35), as well as extrapulmonary inflammatory disease, *e.g.* in lymph nodes, intestine or implant sites of interventional cardiac devices (36–40). Given the high diagnostic accuracy for FDG-PET/CT in substantiating localized inflammation on a whole-body level even in early stages of inflammatory disease (29), and the broad availability of the modality in North and Latin America, Europe, Asia and Oceania with a heterogenous availability in Africa (41), it would be an easy-to-accomplish task to conduct prospective, longitudinal, multicentric studies in COVID-19 patients at different stages of severity, to a) identify extrapulmonary inflammatory disease manifestations and b) search for possible imaging biomarkers of later severe and potentially life-threatening inflammatory reactions, thus possibly identifying individuals in need of specific anti-inflammatory treatment.

While FDG is the most frequently used PET radiopharmaceutical to date, the intrinsic ability of PET to provide tailored targeted molecular imaging has drawn interest from a growing body of medical specialties, including immunology, where radiolabeled monoclonal antibodies (mAbs) and intermediary compounds of the immunological response have been successfully introduced and are summarized under the term Immuno-PET (42–46). Close collaboration between radiopharmacists, radiochemists, clinicians, and nuclear medicine specialists has increased the number and species of radiotracers, with an exquisite pool of established and conceptionalized radiolabeled biomarkers bearing the unique potential of providing primary quantitative data on cellular or even molecular level on a whole-body scale, allowing for a pictorial whole-body cartography of the process under investigation. As current PET imaging has almost completely turned into hybrid imaging, either diagnostic CT or MRI will be acquired along with the PET investigation and thus offers an unparalleled view into the pathophysiological changes associated with an individual course of disease.

In comparison to standard immunological testing, which also provides longitudinal information of inflammatory disease in COVID-19, whole-body imaging adds the anatomical localization of the pathophysiology, which may prove clinically relevant for the assessment, targeted treatment and follow-up of organ-specific disease such as myocarditis, pericarditis, inflammatory bowel disease, pancreatitis and chronic inflammatory disease or vascular inflammation (47).

As each diagnostic receptor binding site also inherently represents a possible target site for newly developed or repurposed therapeutic agents, simultaneous therapeutic application during an imaging study poses an intrinsic displacement challenge for its diagnostic twin, with dynamic imaging allowing for a quantitative, summative monitoring of treatment effect based on cellular level.

The whole potential of PET/CT or PET/MRI, however, can only unfold in an interdisciplinary collaboration of the related disciplines of immunology, virology, clinical medical specialties, pathology and nuclear medicine, and requires a dedicated effort to establish controlled clinical study protocols using a wise selection of rapidly available radiopharmaceuticals (48). Such an effort would allow the medical specialties involved to make best use of the full potential of molecular imaging to identify key pathogenic mechanisms, to quantify disease process and to predict disease outcome, thus facilitating evidence-based medicine in COVID-19.

## Currently Established, Molecular Targets Possibly Suitable for COVID-19

### Chemokines and Chemokine Receptors

An excessive inflammatory response to SARS-CoV-2 at a given timepoint of the disease, often in its later stages, is thought to be a major cause of disease severity and death in patients with COVID-19 and is associated with high levels of circulating cytokines, profound lymphopenia and substantial mononuclear cell infiltration in the lungs, heart, spleen lymph nodes and kidneys, as observed in post-mortem analysis (49).

In the absence of preexisting antibodies to a viral infection, innate immune sensing triggers a pathway of specific immune responses as a first line of antiviral defense. While data on the specific innate immune response to SARS-CoV-2 is still limited, a certain analogy to the well-studied immune responses to other coronaviruses may be expected, where cytosolic retinoic acid-inducible gene I (RIG-I) like receptors (RLRs) and extracellular and endosomal toll-like receptors (TLRs) trigger signaling cascades resulting in the secretion of cytokines (49) and have shown to sit at the crossroads of multiple inflammatory pathways including neuroinflammatory disease (50). Early antiviral defense is mediated by type I/III interferons (IFNs), followed by inflammatory cytokines and chemokines. Immune cell profiling of COVID-19 patients in the recovery stage has shown high expression levels of the chemokine receptor CXCR4 in inflammatory monocytes, possibly indicative for fueling the inflammation during SARS-CoV-2-infection (51). As targeting CXCR4 has been subject to intensive studies due to involvement of the chemokine axis in tumor progression (52, 53), suitable radiopharmaceutical conjugates of CXCR4 as well as potent CXCR4 antagonists have been developed and thoroughly described (54, 55) and thus could be easily repurposed to investigate the cytokine signaling pathway in COVID-19 and monitor possible effects of targeted therapy with CXCR4 antagonists by quantifying the regional level of CXCR4 activity over time. Unlike lab findings mainly reflecting systemic inflammatory activity, molecular imaging has the potential to early identify the involvement of specific organs and monitor their response to therapy.

### ATP-Purinergic Receptor P2X7 Pathway

Another potent activator of the innate immune system related to antiviral response that recently has come into focus, is the purinergic receptor P2X7 (56–58), which is mainly expressed in inflammatory and immune-related myeloid cells – macrophages and microglia. P2X7 receptors are potent stimulants of inflammation, mediating the activation of the NLRP3 inflammasome, as well as cytokine and chemokine release, thus forming a natural target for anti-inflammatory therapy. A number of radiopharmaceuticals selective to the P2X7 receptor have been developed, are meanwhile well established, and have already been put into clinical practice (59). While initially designed to quantify neuroinflammation, they can easily be repurposed for imaging and quantifying localized inflammation at a whole-body scale. As there are also potential P2X7-inhibitors at receptor level, showing anti-inflammatory effects in animal models (60), P2X7-receptor imaging may here form the rationale for directing and monitoring anti-inflammatory therapy in case of regional or systemic involvement in COVID-19.

### Postinflammatory Fibrosis

There is accumulating data that recovery after COVID-19 infection can be associated with persistent organ damage induced by postinflammatory fibrosis (61), and early detection of prolonged fibroblast activation related to inflammation could warrant tailored therapies directed at mitigation of fibrosis and an underlying inflammatory process. Molecular imaging here could play a critical role for understanding the extent of systemic disease and, by visualizing, help to direct therapy and follow up therapy response. One out of a number of possible molecular targets that have gained considerable interest most recently, are the integrins, which derive their name from which derive their name from their role of integrating the extracellular matrix with the insoluble cellular cytoskeleton. The field of integrin research was advanced enormously by the detection of the crystal structure of the extracellular domain of *α*v*β*3 in 2001 (62). This structure shows that the globular head of the integrin is made up of a seven-bladed *β*-propeller contributed by the *α*-subunit and the A-domain from the *β*-subunit together with an immunoglobulin fold, made up of polypeptide sequences from either side of the *β* A-domain. The integrin stalks are folded into three *β*-sandwich domains in the *α* and four epidermal growth factor (EGF)-like repeats in the *β*-subunit. The ligand-binding specificity of integrin molecules is determined by the particular *α*–*β* combination and consequently determines the adhesion specificity of different cell types. Recent research has successfully led to radiolabeled *α*v*β*6-binding peptides, which are targeting an epithelium-specific cell surface receptor that is low or undetectable in healthy adult epithelium but upregulated in select injured tissues (63–65), where it has successfully been shown by immunohistochemical analysis of biopsy specimens, that tissues showing high uptake on 18F- *α*v*β*6-BP PET/CT in patients with cancer indeed correlated with high expression of integrin *α*v*β*6 for tissues (NCT03164486) (64). Following the call to repurpose well characterized imaging biomarkers for COVID-19 research (66), the same group recently proposed to extend the use of the demonstrated integrin avb6 imaging agent 18F-*α*v*β*6-BP to assess lung damage in patients after severe acute respiratory syndrome coronavirus 2 (SARS-CoV-2) infection in a first-in-human study (67). In their preliminary study, the authors were able to demonstrate a correlation of integrin *α*v*β*6-targeted 18F-*α*v*β*6-BP PET with lung damage identified by CT. The authors concluded *in-vivo* integrin imaging to be a promising strategy to detect and monitor the development and progression of lung fibrosis after SARS-CoV-2 infection, with a better understanding of the nature of the tissue remodeling and progression in recovering patients over time being the key for developing therapeutic strategies to prevent irrecoverably disease.

Another key protein for activation of the fibroblast cascade, leading to destructive organ fibrosis, is the fibroblast activation protein FAP (68), which has been studied for almost three decades. As it has gained special attention as a highly expressed marker in the vast majority of epithelial tumors, considerable amount of research has been put into developing FAP-radioligands, resulting in a number of radiolabeled FAP-antagonists suitable for whole-body PET imaging (69). One of those radiolabeled imaging agents, 18-F-FAPI has just entered clinical oncologic imaging on the base of large-scale clinical trials, with the potential of becoming the next most used PET imaging agent besides FDG, as it has been shown to prove diagnostic information for a multitude of different tumor entities.

With novel FAP antagonists showing promising first results to mitigate FAP induced fibrosis (70), the repurposing of FAP-directed radiopharmaceuticals currently under extensive investigation for oncology may open up a new door in directing protective or restorative therapy to treat COVID-19 associated sequelae.

### ACE-2-Receptor and ATR1-Receptor Imaging

Other, still more experimental radiopharmaceuticals which would prove useful in COVID-19 infection focus on directly targeting the entry receptor for SARS-CoV-2, the angiotensin-converting enzyme 2 (ACE-2)-receptor, involved in cellular internalization of SARS-CoV-2 (71, 72) and, downstream, the type 1 angiotensin-II-receptor ATR1 (*e.g.* KR31173). Numerous reports have linked tissue specific ACE-2 expression to organ injuries by ACE-2-mediated viral homing and attachment to organ-specific cells, as well as to the role of ACE-2 bearing macrophages potentially serving as trojan horse, triggering pulmonal anchoring and migration out of the lung to other tissues alike (73). Whole-body ACE-2-receptor imaging would therefore provide a unique means to survey entry-points as well as immunoreactivity during the course of COVID-19 infection.

Radiolabeling of ACE-2-receptor antagonists has been achieved for receptor autoradiography protocols (74), and could serve as starting point for a future PET tracer development that would be able to provide quantitative data on receptor based virus entry load *in-vivo*. Preclinical imaging of ATR1 has also been successfully achieved using ^11^C- or ^18^F-labeled compounds and preclinically available receptor antagonists (75), which could be used to better define and verify a point of pharmaceutical intervention.

### Role of Cyclooxigenase-2 in COVID-19 Related Inflammatory Reaction

COX-2 is unexpressed under normal conditions in most cells, and expression is stimulated *via* up-regulation of prostaglandins during inflammation of various types. Non-steroidal anti-inflammatory drugs (NSAIDs) inhibit prostaglandin production, Cyclocoxigenase-1 (COX-1) and COX-2. NSAIDs selective for inhibition of COX-2 are commonly prescribed for the control of fever and other symptoms in COVID-19 and recently have come under scrutiny for anecdotal reports of worsening the course of disease. In the current discourse of controversial concepts of biological plausibility that possibly leads to contradictory concepts of recommendations (76), quantitative imaging of COX-2-involvement using established COX-2-inhibitory radiopharmaceuticals (77) would lead the way to provide evidence for general therapeutic guidelines or could even inform individualized future treatment options.

### Cell Tracking of CD-8+ T-Lymphocytes

T-lymphocytes and natural killer (NK)-cells play critical roles in viral clearance during respiratory infections. Recent reports of decreased ratio of CD8+ T cells in COVID-19 patients may implicate a role of CD8+ T cells in virus clearance (51), mostly at a later timepoint of the disease. CD8+ T-lymphocytes are preferred immune cells in cancer immunotherapy, and extensive research already has gone into PET imaging of cytotoxic human T cells, recently resulting in first-in-human imaging studies of anti-CD-8 imaging in patients with solid tumors (78). The application of novel radioligands with radioactive half-time of more than 72 h allows for the concept of *in-vivo* cell tracking of CD-8+ T-lymphocytes, which for clinical application in solid malignancies currently is in a Phase-II clinical trial (79). *In-vivo* cell tracking of CD-8+ T-lymphocytes would therefore be available in dedicated research centers and could contribute to monitoring the course of immune defense during COVID-19 infection and possibly, response to convalescent plasma therapy (80) or early immunization trials. They also could prove helpful in tracking a possible involvement of aberrant activation of CD-8+ T-lymphocytes in multi-organ damage including cardiac injury, which will be of increasing clinical importance with a steadily rising number of reports on multi-organ involvement in COVID-19.

## Future Perspectives for Depicting Events at Earlier Time Points of COVID-19

Proinflammatory chemokine receptor 2+ (CCR2+) macrophages are important mediators of inflammation during the early course of COVID-19 and are therefore a potential target of interest for quantitation of localized inflammation in the beginning of infection with SARS-CoV-2. CCR2-binding peptides have been labeled with ^64^Cu for use as PET radiotracer (^64^Cu-DOTA-ECL1i) and have been successfully evaluated in a preclinical mouse model of lung inflammation (81, 82). The very rapid blood clearance of ^64^Cu-DOTA-ECL1i, however, may possibly limit its practical use in human studies, and further research may be necessary to adapt this promising PET probe to clinical needs in imaging early course of inflammatory disease in humans.

Additionally to CCR2+monocytes, according to, IL-6 could also be a target for PET probes at earlier time points of COVID-19. There is, however, currently no conceptualized radiotracer targeting IL-6, warranting further radiopharmaceutical research to fill this gap.

## Summary

Due to the still unfolding character of the COVID-19 pandemic, only the lowest level of evidence for medical imaging (level V out of five levels of evidence-based medicine criteria) is available, with a consensus statement reached by a multidisciplinary expert panel just recently jointly published by the American College of Chest Physicians and the Radiological Society of North America and a rapid advice guide published by the World Health Organization (WHO) in June 2020. According to their statements, chest-CT is regarded appropriate in patients with COVID-19 and worsening respiratory status (83), and for symptomatic patients with suspected COVID-19, when rt-PCR testing is not available or delayed or when initial rt-PCR testing is negative, but a high clinical suspicion of COVID-19 persists (84). With only limited experience for other imaging modalities such as Ultrasound, MRI and FDG-PET/CT, there are no evidence based criteria yet allowing to recommend their use in COVID-19 on a routine base.

Molecular imaging, however, offers a potential clearly exceeding the obvious primary clinical goal, to establish a correct diagnosis: it offers tailored means to identify and quantify on cellular and molecular levels important elements of the cascade of immunological and immune-mediated events triggered by the entrance of SARS-CoV-2 into the human body and to ultimately predict at early timepoints the course of disease in an individual patient, which would form the rationale for a patient-centric, tailored therapeutic approach.

Limiting an early success of molecular imaging would be that clinical manifestations of COVID-19 are complex and not all patients are suffering the same symptoms—which would imply that clinicians and immunologists should widely use molecular imaging for COVID-19 and systematically investigate patients, defining and using those molecular targets that are most promising for understanding the course of disease and tailoring timely therapy to prevent sequelae and death from this still devastating disease, taking into account as much of interdisciplinary collaboration as possible and necessary.

While costs may also be considered a limiting factor for wide application of molecular imaging, cost calculations would have to take into account the potential of costs saved by preventing more severe courses of disease, chronic disease and possible early death. Considering that molecular imaging has been shown to be cost-effective in a variety of cancer conditions (85–87), it is conceivable that this might equally be the case for COVID-19, where life expectancy and life quality without the event of infection would be unimpaired. Allocation of research funds would thus follow the same principles as in cancer research.

Undoubtedly, there is an urgent need for higher level of evidence for medical imaging in COVID-19 and, subsequently, for derived decisions on treatment approaches. As controlled studies on COVID-19 patients will have to be performed in an interdisciplinary setting and precautions to comply with for medical procedures in these patients are high, any imaging study should be designed in a way to generate the most comprehensible data possible. Nuclear medicine procedures using PET/CT and PET/MR are elegantly combining imaging data from two modalities in a single imaging session, thus reducing the number of patient visits and contact time under extensive precautions. Besides providing new pathophysiological insights of immune response in COVID-19 using molecular probes, any PET/CT or PET/MR performed in these patients will also provide fully diagnostic CT or MRI exams at the same time, thus being able to answer clinical questions in need, and according to current evidence. Additionally, as PET/CT and PET/MR by design are intended to cover a whole-body imaging field, nuclear medicine will be able to shed light on the increasing number of extrapulmonary organ involvement that we are just beginning to fully recognize. With the glucose analog on FDG being the most available routine tracer for PET imaging, an unspecific but highly sensitive probe for localized or generalized inflammatory disease is readily and immediately available, and given the improved sensitivity of the current generation of PET/CT and PET/MRI scanners, these procedures can be safely performed even in the youngest of our patients and in the worst of their clinical conditions.

Tailored radiopharmaceuticals beyond FDG that already have been established and thus can be made available with modest effort will, on a short time-scale, allow to conduct controlled and prospective studies of various levels on interaction of SARS-CoV-2 related immunopathomechanisms, to better understand when and where detrimental processes are being activated. This would help to early diagnose critical organs at risk of inflammatory destruction and to potentially guiding the observer on disease-directed pharmacologic interventions according to receptor status, biochemical status or receptor ligand status.

**Figure 1:**
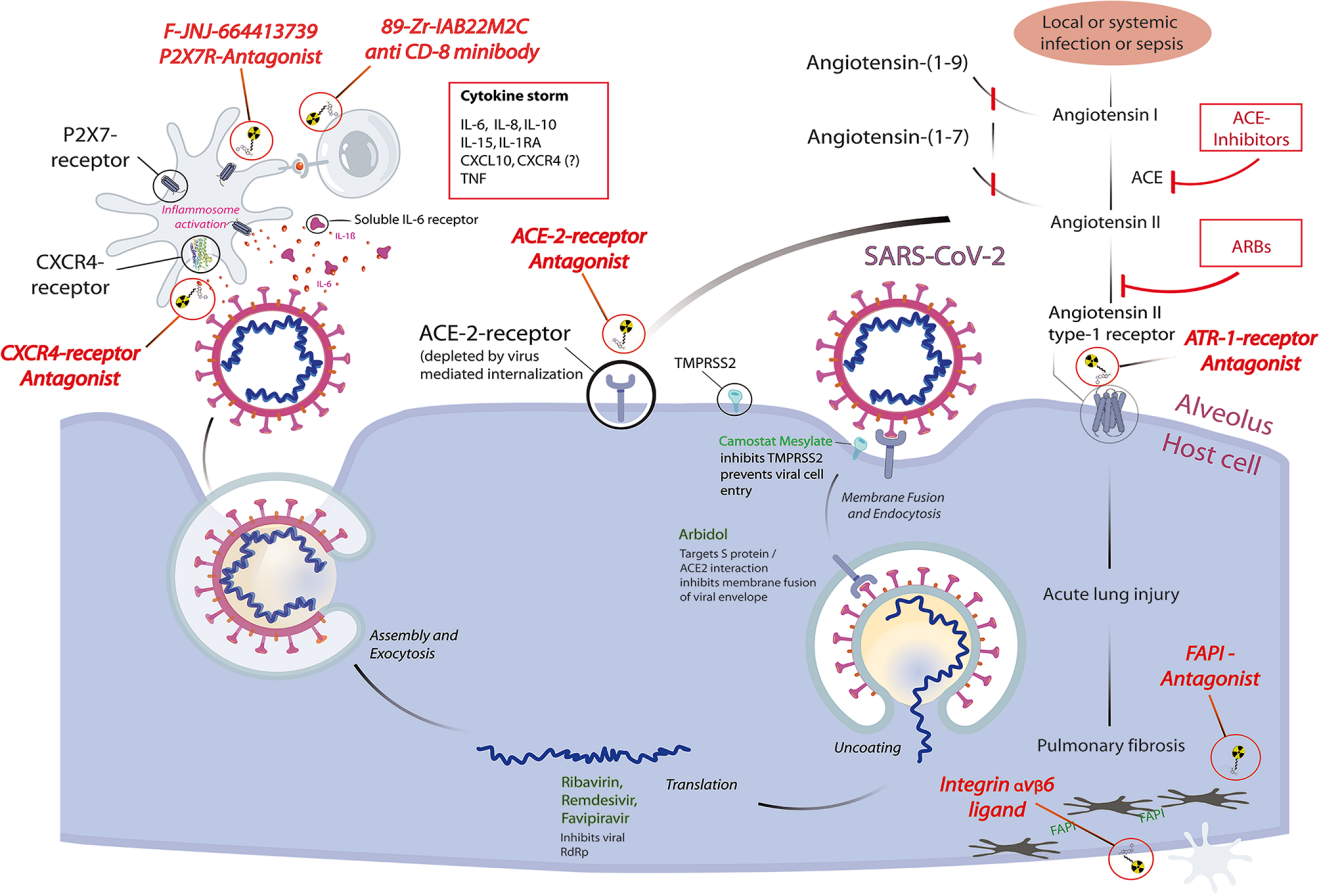
Pictorial review highlighting selected molecular imaging targets for pathophysiological features of coronavirus disease 2019 (COVID-19) and their relation to current concepts of therapeutical interventions, based on a schematic representation of the reproduction cycle of SARS-CoV-2 at the alveolus–host cell interface. Radiopharmaceuticals (red italic) are depicted at their specific binding sites: receptor antagonists binding to their respective receptors, integrin ligands and FAPI-antagonists schematically binding to their respective target structures. Intracellular therapeutic interventions (green) and systemic interventions (red) are shown for their possible downstream effects that will influence the dependent functional imaging principle. Each diagnostic receptor binding site also represents a possible therapeutic target site, thus any therapeutic use of receptor antagonists intrinsically forms a displacement challenge for its diagnostic twin, allowing a direct quantitation of localized therapeutic receptor binding.

Institutions providing one of the most advanced whole-body PET scanners like the uEXPLORER at UCDavis or the PennPET Explorer might spearhead clinical studies by their unique capability of fast and comprehensive whole-body dynamic and quantitative imaging and of whole-body acquisition times in the range of seconds. PET/MRI using dedicated tracers could prove to be helpful in cases of occult myositis, endocarditis, myocardial inflammation, or myopathy, as well as CNS involvement. A pictorial review of selected, possible imaging targets and their relation to current concepts of therapeutical interventions is given in **Figure 1**.

And finally, if further, specific targets of interest are discovered by clinicians, appropriate biochemical markers and radiopharmaceuticals can be tailored to address the most imminent needs.

The examples on radiopharmaceuticals chosen here are not meant to be comprehensive. They are meant to give a perspective and to exemplify how certain steps of viral interaction within the human body and its immune response can be quantitatively and longitudinally assessed, potentially directly guiding specific treatment based on evidence, using existing and well described radiopharmaceuticals that are easily to be repurposed. The examples are also meant to stimulate the scientific community’s potential to contribute to one of the biggest challenges in modern medicine and to plan and conduct multicentric trials and to share what it needs for making novel radiopharmaceuticals accessible at an accelerated time scale.

## Conclusion

Molecular imaging repurposing molecular probes already established in nuclear medicine have the immediate perspective of facilitating evidence based medicine in COVID-19 by providing *in-vivo* quantitative measures of molecular and immunological mechanisms on a whole-body scale. To fulfill this goal, immunologists and clinicians alike should widely use molecular imaging as a longitudinal measure in future clinical trials as well as in controlled patient cases. A prospective build-up of a longitudinal molecular imaging database for COVID-19, including pathological findings, lab measures, and clinical follow-up will be key to identifying imaging biomarkers and other predictive measures of prognostic significance.

## Data Availability Statement

The original contributions presented in the study are included in the article/supplementary material. Further inquiries can be directed to the corresponding author.

## Author Contribution

FJ, AM, FW, and TS contributed to the conception and design. FJ wrote the first draft of the manuscript. AM, FW, and TS performed review and revision of the manuscript. FJ prepared the illustration. All authors contributed to the article and approved the submitted version.

## Conflict of Interest

The authors declare that the research was conducted in the absence of any commercial or financial relationships that could be construed as a potential conflict of interest.

## Abbreviations

COVID-19, Coronavirus disease 2019; SARS-CoV-2, Severe acute respiratory syndrome coronavirus 2; ARDS, Acute respiratory distress syndrome; CT, Computed tomography; AI, Artificial intelligence; FDG, Fluordeoxyglucose; PET, Positron emission tomography; MRI, Magnetic resonance imaging; CNS, Central nervous system; RIG-I, Cytosolic retinoic acid-inducible gene I; RLRs, RIG-I like receptors; TLRs, Toll-like receptors; IFNs, Interferons; CXCR4, C-X-C Motif chemokine receptor 4, also: chemokine-receptor type 4; ATP, Adenosinetriphosphate; P2X7, ATP-purinergic receptor P2X7, also: P2X purinoceptor 7; FAP, Fibroblast activation protein; ACE-2, Angiotensin-converting enzyme 2; ATR1, Angiotensin-II-receptor type 1; COX-2, Cyclooxigenase-2; NSAIDs, Non-steroidal anti-inflammatory drugs; COX-1, Cyclooxigenase-1; CD-8+, Cluster of differentiation 8 antigen positive; NK-cells, Natural killer cells; uEXPLORER, Ultra-High-Resolution total-body digital PET/CT with 194 cm axial field of view; UCDavis, University California, Davis; PennPET, Pennsylvania University whole-body digital PET/CT with 64 cm axial field of view; WHO, World Health Organization.
